# Arterio-Venous Fistula: Is it Critical for Prolonged Survival in the over 80's Starting Haemodialysis?

**DOI:** 10.1371/journal.pone.0163487

**Published:** 2016-09-29

**Authors:** Adam D. Jakes, Poonam Jani, Victoria Allgar, Archie Lamplugh, Ahmed Zeidan, Sunil Bhandari

**Affiliations:** 1 Hammersmith Hospital, Imperial College Healthcare NHS Trust, NHS Trust, London, United Kingdom; 2 King’s College Hospital NHS Foundation Trust, Denmark Hill, London, United Kingdom; 3 University of York, York, United Kingdom; 4 Department of Renal Medicine, Hull Royal Infirmary, Hull and East Yorkshire Hospital NHS Trust, Kingston upon Hull, United Kingdom; 5 Hull York Medical School, East Yorkshire, United Kingdom; University of Florida College of Medicine, UNITED STATES

## Abstract

**Background:**

Dialysis in elderly patients (>80-years-old) carries a poor prognosis, but little is known about the most effective vascular access method in this age group. An arteriovenous fistula (AVF) is both time-consuming and initially expensive, requiring surgical insertion. A central venous catheter (CVC) is initially a cheaper alternative, but carries a higher risk of infection. We examined whether vascular access affected 1-year and 2-year mortality in elderly patients commencing haemodialysis.

**Methods:**

Initial vascular access, demographic and survival data for elective haemodialysis patients >80-years was collated using regional databases. A cohort of conservatively managed patients was included for comparison. A log-rank test was used to compare survival between groups and a chi-square test was used to compare 1-year and 2-year survival.

**Results:**

167 patients (61% male) were included: CVC (101), AVF (25) and conservative management (41). Mean age (median) of starting haemodialysis (eGFR ≤10mL/min/1.73m^2^): CVC; 83.4 (2.3) and AVF; 82.3 (1.8). Mean age of conservatively managed patients reaching an eGFR ≤10mL/min/1.73m^2^ was 85.8 (3.6). Mean (median) survival on dialysis was 2.2 (1.8) years for AVF patients, 2.1 (1.2) for CVC patients, and 1.5 (0.9) for conservatively managed patients (p = 0.107, controlling for age/sex p = 0.519).

1-year and 2-year mortality: AVF (28%/52%); CVC (49%/57%), and conservative management (54%/68%). There was no significant difference between the groups at 1-year (p = 0.108) or 2-years (p = 0.355).

**Conclusion:**

These results suggest that there is no significant survival benefit over a 2-year period when comparing vascular access methods. In comparison to conservative management, survival benefit was marginal. The decision of whether and how (choice of their vascular access method) to dialysis the over 80s is multifaceted and requires a tailored, multidisciplinary approach.

## Introduction

Renal replacement therapy is indicated when kidney function declines to the point that retention of waste substances leads to significant clinical symptoms and signs. The estimated glomerular filtration rate (eGFR) at which dialysis is needed is variable throughout the world but is typically ≤10ml/min/1.73m². The mean age of starting haemodialysis in the UK is 66.9 years, however with an aging population the number of dialysis-dependent patients ≥80-years continues to increase. There are approximately 2,700 patients ≥80-years requiring dialysis/million age-related population in the UK [1]. These patients are much frailer than typical dialysis-dependent patients, with multiple comorbidities and reduced life expectancy. In an elderly dialysis population ≥80-years, the one-year survival rate was 54%, compared to 80% in those 70–74 years. Survival was also directly proportional to the number of comorbidities [2]. Murtagh *et al*. found that one and two year survival rates in patients ≥75 years were 84% and 76% respectively in the dialysis group compared with 68% and 47% respectively in patients treated conservatively [3]. However, this survival advantage was lost in those with a large number of comorbidities, particularly ischaemic heart disease.

Renal replacement therapy requires vascular access that is ideally both high-flow and low resistance. An arteriovenous fistula (AVF) is time-consuming (requiring maturation time of up to six weeks), and initially expensive due to the requirement of theatre resources and surgical creation. Despite the lower maintenance costs, there is a significant risk of primary failure as elderly patients often have poor-calibre blood vessels [4]. Contrastingly, a central venous catheter (CVC) is initially cheaper, with the majority inserted under local anaesthetic in a day-case theatre; however the use of similar staff and equipment together with subsequent line complications (e.g. infection and bleeding) offset these costs. Accordingly, the cost of vascular access at one year has been estimated as $7989 Canadian dollars for an AVF compared to $9180 for a CVC [5].

The type of haemodialysis access affects survival of patients commencing dialysis, with nearly a 50% increase in mortality, particularly infection-related deaths, when CVCs are used compared to AVFs [6]. An AVF is superior to a CVC due to its potential survival benefits, as advocated by the National Kidney Foundation Dialysis Outcomes Quality Improvement (NFK-KDOQI) [7] and UK Renal Association [8]. The questions remain whether the insertion of a CVC is as efficient at facilitating haemodialysis via a fistula in patients ≥80-years and does not increase mortality.

We compared the 1 and 2-year survival of patients ≥80-years undergoing haemodialysis using either a CVC or an AVF as their initial vascular access. A third cohort of conservatively managed patients was included for comparison against these two groups.

## Methodology

A retrospective study of patients ≥80-years who started chronic haemodialysis at Hull and East Yorkshire Hospitals NHS Trust between 2000–2011 were identified (those patients requiring emergency dialysis were excluded). Demographic data at the time of starting dialysis, aetiology of renal disease, initial vascular access (AVF or CVC), comorbidities and cause of death (where appropriate) were recorded. Patients who received arteriovenous synthetic grafts (an uncommon form of access) or renal transplants were excluded. Dialysis regimen was consistent with at least four hours three times a week aiming to achieve blood pump speeds of ≥350ml/min and a clearance as measured by urea reduction ratio of at least 65%. A third group of elderly patients, also ≥80-years, who had elected to receive maximal conservative management, was included. The date corresponding to an eGFR ≤10ml/min/1.73m^2^ was noted so a reasonable comparison could be made between conservative and dialysis patients to minimise the possibility of lead-time bias. Survival data from all cohorts was collated using regional databases and analysed at the point of commencing dialysis and death (if occurred), up to 2 years. These databases contained information for submission to the UK Renal Registry which has continued to evolve over the years, collecting more detailed information more recently. This study formed part of service improvement (quality improvement project as specified by the Royal Colleges in the UK), therefore ethical approval was not required but trust approval was provided. In addition all patients entering the dialysis program agree to have their data used to monitor quality and for research.

The aetiology of renal failure was divided into subcategories: diabetes, hypertensive and vascular disease, glomerulonephritis, unknown and other (glomerulosclerosis, interstitial nephritis, obstructive nephropathy, pyelonephritis and polycystic kidney disease). These categories were chosen due to their common use within the literature. The cause of death was also documented as infective, cardiovascular, malignancy, dialysis withdrawal, unknown or other. Cardiovascular death was defined as death due to an acute myocardial infarction, atherosclerotic heart disease, cardiomyopathy (uraemic or hypertensive), arrhythmia, cardiac arrest, sudden death or stroke.

All patients were assessed on an intention to treat basis, relying on evidence that they were followed up until either death, renal transplantation, or lost to follow up.

### Statistical analysis

All statistical analyses were completed using SPSS. Dependent variables are expressed as means, interquartile ranges and standard deviations. Independent variables are expressed as percentages of total study population. In comparing groups of three independent groups, a non-parametric test was used. Survival was analysed using the Kaplan-Meier method. A log-rank test was used to compare survival between the three groups and mean (standard error (SE), 95% confidence intervals) and median (SE, 95% confidence interval) are quoted for survival data. Cox regression was used to control for age and gender. One year and two year survival was calculated (95% confidence intervals) and a chi-square test was used to compare one year and two year survival. A p-value of <0.05 was considered statistically significant. Analysis was undertaken on STATA/SE (V14, Timberlake Consultants Limited).

The study was granted approval from the NHS Hospital Trust Research and Development Committee. Principles of good clinical practice (GCP) were followed and to ensure confidentiality all data was anonymized. All patients entering the dialysis service verbally consent to the use of their data for research and analysis.

## Results

167 patients were included; 101 initially dialysed using a CVC, 25 dialysed via AVF and 41 treated conservatively. 102 (61%) were men and 21 (13%) were diabetic (44 unknown). The mean age of starting dialysis was 83.3 (SD: 2.99, range: 80–96) years. This was similar in both CVC and AVF groups; 82.9 (2.3) and 81.7 (1.9) years respectively. The mean age of conservative patients reaching an eGFR ≤10ml/min/1.73m^2^ was 85.3 (3.7) years.

Table 1 demonstrates patient baseline characteristics, along with comparing those with CVC or AVF access. Comorbidities were similar in both groups except for ischaemic heart disease, with a much lower incidence in those dialysed via an AVF compared to a CVC (12% vs. 32%). This was mirrored in cause of death, which demonstrated a higher cardiovascular cause of death in the CVC group. The aetiology of renal failure was also similar in both groups, with more than one third of the cases the aetiology being unknown. Most patients had at least 2 comorbidities.

**Table 1:** Patient Baseline Characteristics.

Initial Access	Total	CVC	AVF
Number	n = 126	n = 101	n = 25
**Male Sex**	78/126 (62%)	63/101 (62%)	15/25 (60%)
**Mean age of commencing dialysis in years (SD)**	83.3 (2.9)	82.9 (2.3)	81.8 (1.9)
**Ischaemic Heart Disease**	35/124 (28%)	32/99 (32%)	3/25 (12%)
**Malignancy**	21/124 (17%)	17/99 (17%)	4/25 (16%)
**Diabetes**	21/123 (17%)	18/98 (18%)	3/25 (12%)
**Aetiology number (%)**			
**Diabetes**	7 (6%)	5 (5%)	2 (8%)
**Vascular Disease**	17 (14%)	14 (14%)	3 (12%)
**Obstructive**	13 (10%)	10 (10%)	3 (12%)
**Glomerulonephritis**	11 (9%)	11 (11%)	0 (0%)
**Unknown**	55 (44%)	41 (40%)	14 (56%)
Other*	23 (18%)	20 (20%)	3 (12%)

Data is expressed as mean (standard deviation) for age or as a number and percentage (%) of patients. Abbreviations: CVC–Central venous catheter, AVF–Arteriovenous fistula.

*Other includes those with glomerulosclerosis, polycystic kidney disease, interstitial nephritis, and pyelonephritis.

At 3 months, 23/25 AVF patients were alive but 4 had fistulae-failure requiring dialysis via CVC. At 6 months, 19/25 AVF patients were alive, with one further fistula-failure. Sixty percent of patients with fistula-failure were female.

Twenty patients were still alive at analysis (all surviving >2 years).

**Fig 1:**
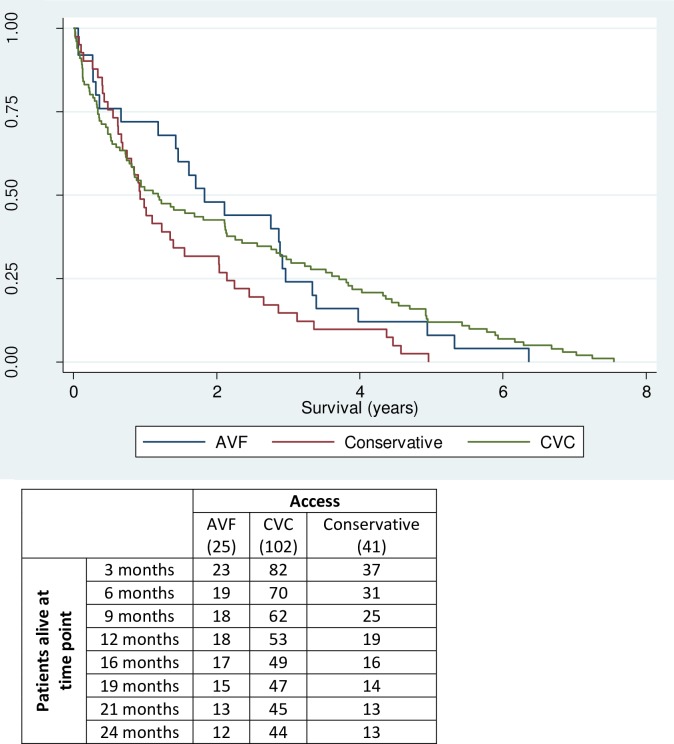
Unadjusted survival curve comparing the AVF, CVC and conservatively managed patients reaching an eGFR < = 10ml/min/1.73m^2^.

Fig 1 and Table 2 shows that there was no statistically significant difference between the groups (p = 0.107). The mean (median) survival on dialysis was 2.2 (1.8) years for AVF patients, 2.1 (1.2) for CVC patients, and 1.5 (0.9) for conservatively managed patients reaching an eGFR ≤10ml/min/1.73m^2^. After controlling for age and sex, there was still no statistically significant difference (p = 0.519).

**Table 2:** Dialysis and Survival Data.

Initial Access	Total	CVC	AVF	Conservative Patients	P value
Number	n = 126	n = 101	n = 25	n = 41	
**Mean (SE) survival in years (95% CI)**	2.1 (0.2)	2.1 (0.2)	2.2 (0.3)	1.5 (0.2)	0.107
	(1.8, 2.6)	(1.7, 2.6)	(1.5, 2.9)	(1.1, 1.9)	
**Median (SE) survival in years (95% CI)**	1.4 (0.3)	1.2 (0.3)	1.8 (0.4)	0.9 (0.1)	
	(0.8, 2.0)	(0.6, 1.8)	(1.0, 2.6)	(0.7, 1.1)	
**One year mortality**	59 (44%)	49 (49%)	7 (28%)	22 (54%)	0.108
**Two year mortality**	71 (56%)	58 (57%)	13 (52%)	28 (68%)	0.355
**Cause of death**					
Infective	17 (14%)	13 (13%)	4 (16%)		
Cardiovascular	28 (21%)	26 (26%)	2 (8%)		
Malignancy	4 (3%)	2 (2%)	2 (8%)		
Unknown	37 (29%)	29 (29%)	8 (32%)		
Withdrawn	19 (11%)	15 (15%)	4 (16%)		
Other	7 (12%)	7 (7%)	0 (0%)		
*Alive at analysis*	14 (16%)	9 (9%)	5 (20%)		

Data is expressed as mean and median (standard error (SE), 95% confidence intervals) or number (percentage) of patients for survival. Cardiovascular death was defined as death because of acute myocardial infarction, atherosclerotic heart disease, cardiomyopathy, arrhythmia, cardiac arrest or stroke.

Table 2 demonstrates that there was no survival difference between patients with either vascular access method, and patients who elected conservative care management.

Table 2 shows that the mortality (median) of patients using a CVC at 1 year was 49% (39%, 59%), in contrast to patients with an AVF, where mortality was 28% (12%, 49%) and in conservatively managed patients this was 54% (37%, 69%). There was no statistically significant difference between the three groups (p = 0.108). The mortality of patients using a CVC at 2 years was 57% (47%, 67%) compared to patients with an AVF, where mortality was 52% (31%, 72%) and for conservatively managed patients this was 68% (52%, 82%). There was no significant difference between the three groups (p = 0.355).

The majority of patients died of cardiovascular (28; 22%) and infective (17; 14%) causes, with the majority from the CVC group; 26/28 and 13/17 respectively. Fourteen (11%) patients withdrew from dialysis and subsequently died. No data regarding aetiology or cause of death was available for patients treated conservatively due to death at home or in a community setting.

## Discussion

Recognised national [8] and international [7] guidelines recommend the use of an AVF in long-term haemodialysis. The age of patients starting dialysis is gradually increasing, and little guidance has been published regarding the type of vascular access in patients≥80-years. Recent studies argue that while specific subgroups in the haemodialysis population exist, use of fistulas and grafts at time of dialysis initiation is not of proven statistical benefit to survival. Elderly haemodialysis patients with comorbidities still appear to benefit from the use of fistulas and grafts [9]. However, fistula first does not seem to be clearly superior to graft placement first in the elderly, because each strategy associates with similar mortality outcomes in octogenarians and nonagenarians [10].

Our aim was to assess the correlation between vascular access (AVF and CVC; very few AV grafts are inserted in our centre and in the UK) and survival in this population, after accounting for age and sex. In our study the mean age of starting haemodialysis was 83 years. The median age within our local dialysis population (incidence 2012; 90/million population/year) is similar to the national average 64.1 vs. 64.9 years respectively [1].

The mortality at one year in patients with an AVF compared to a CVC (28% vs. 49%) was not statistically significant (p = 0.108), perhaps in part due to the size of the cohort studied. This trend towards a difference was not present at 2 years (52% vs. 57%) (p = 0.355) suggesting that the “potential” survival advantage within the first year from having an AVF becomes negligible at two years. An intriguing finding was the mortality in the conservative care group, which was not dissimilar from the haemodialysis groups at 1 year. This may be an important finding in considering the selection of elderly patients for dialysis and whether this represents a beneficial intervention. However without more measures of quality of life and detailed comorbidity data this can only be seen as a speculation warranting further clinical study.

Patients with a CVC are at a greater risk of early complication and death from infection, with an incidence of 2–6 infections per 1000 catheter days [11] and a mortality of 3.4% compared with 0.8% in patients using an AVF [12]. More recently a small single-centre study reported 0.55 bloodstream infections per 1000 catheter days in patients aged ≥75-years [13]. A relatively high infection rate was seen in our study, with 13/17 deaths from infection being in patients with a CVC. In addition to the higher mortality due to infection, there is an incurred annual cost with CVC-induced infections. Haemodialysis therapy alone regardless of vascular access method can cost up to £35,000/year [14], however additional costs can be incurred for each patient hospitalised with a CVC-associated bacteraemia. The number of patients dialysed via an AVF in our study was small (25), indicating that current practice may not favour this method in patients≥80-years. It is interesting to note that only 2 (8%) patients died within 30 days of fistula creation, indicating a low post-surgical mortality. We suggest that the trend in survival advantage at 1 year with haemodialysis via an AVF compared to a CVC may be due in part to lower early infection rates. This apparent advantage is lost after 2 years. This is consistent with published literature suggesting that the risk of infection is highest in the first 6 months after vascular access is inserted [15].

It is interesting that although an AVF is widely accepted to be the gold standard of vascular access, the majority of our elderly patients were dialysed via a CVC, not an AVF (grafts as a form of vascular access are uncommon in the UK with only 1% use in our unit). The fact that these patients were ≥80-years with multiple comorbidities may have discouraged surgeons from performing fistula surgery. It is also noteworthy that the incidence of ischaemic heart disease in those who received an AVF was much lower, suggesting this may be one such marker surgeons use for patient selection. The ‘Fistula First, Catheter Last’ campaign was started by a large network of healthcare societies and insurance companies in the USA after AVF were considered the gold standard for haemodialysis vascular access. Fistula success rates are associated with the mechanical integrity of vessels, which in part can be assessed through vascular imaging. Failure rates of AVFs are about 0.2 events per patient/year [16]. However, Swindelhurst *et al*. [17] demonstrated an increase in maturation failure rates in patients ≥65-years old compared to those <65-years, together with more overall failures (13% and 8.9% respectively) associated with occlusion or thrombosis. Al-Jasishi *et al*. [18] report even higher rates of primary failure in patients both below (23%) and above (37%) the age of 65 years old using an AVF. These findings are unsurprising due to higher rates of atherosclerosis, diabetes and hypertension [19, 20]. Altered blood flow dynamics associated with ageing or pathology and reduced vessel compliance will be present in an elderly population [21]. This together with intra-vascular pathology will negatively impact the desired high-flow, low resistance system. The primary failure rate of an AVF in our study at 3 and 6 months was 4 (16%) and 5 (20%). Sixty percent were female, which has been previously reported by Bel’eed-Akkari *et al*. [4]. They also reported similar secondary failure rates, however only one patient in our study had a second AVF, which was used up until their death. It is noted however; that a large, retrospective cohort study concluded that there was no difference in fistula patency with age and advised that an AVF should be offered to patients ≥80-years where appropriate [22].

Evidence is starting to emerge that deviates away from the ‘fistula first’ guidance, especially for a subset of patients, notably the elderly. A recent study concluded that a patient-centred approach to the choice of dialysis access, incorporating recent evidence and patient preference may be preferred [23]. Gomes *et al*. [24] noted that the gold standard fistula may not be the most suitable option for every patient, and patient choice may be more optimal.

Interestingly a recent study from Verberne *et al*. [25] found that in 200 patients > 70-years who had dialysis versus 107 who were treated with conservative management, there was a higher overall median survival for patients treated with dialysis from the time of treatment decision– 3.1 years (IQR 1.5–6.9) for dialysis group in comparison to 1.5 years (IQR 0.7–3.0) for the conservative group. They also noted that this apparent advantage was significantly reduced in those patients with additional cardiovascular comorbidity or multiple comorbidities. However when they examined patients who were ≥ 80-years this survival advantage was lost. The median survival was 2.1 years (IQR 1.5–3.4) compared to 1.4 years (IQR 0.7–3.0). Finally a recent systematic review confirmed this finding of a similar survival in dialysis versus conservative groups [26].

The combined use of risk factors to predict morality in elderly patients is relatively unknown. We have performed a further examination of the hospital patient record system and collected data on haemoglobin concentration and albumin levels at the most recent time point prior to dialysis initiation; this indicated that the mean values for haemoglobin were 95.6g/L (CVC) versus 108.4g/L (AVF); (p = 0.18) and for serum albumin 29.3g/L versus 34.9g/L (p = 0.007). This must be again viewed with caution in view of the small numbers. Recently Bansal and colleagues [27] have shown that in their risk model of elderly patients serum albumin was not useful in predicting death. Biomarkers such as FGF-23 and N terminal pro brain naturetic peptide (NT pro-BNP) may improve predictions of mortality but are not easily available and in the latter case is affected the by level of renal dysfunction in addition to myocyte stress and stretch [28]. Hence in our study it could be argued that further evaluation of these and other biomarkers may be interesting but perhaps not valid. In addition, our study lacks a detailed evaluation of characteristics that are associated with aging and are predictive of mortality such as mobility, frailty or functional status. Data on the Karnofsky and Stoke scores were incomplete in our population with data available on 44 patients. Comparison of Karnofsty scores gave similar values—69.9 for CVCs and 72.7 for AVF’s. However, future development of risk models may help in assisting one in making clinical decisions regarding vascular access and dialysis.

Patients ≥80-years tend to be much frailer, with poor life expectancy compared to their younger counterparts. Quality of life is an important aspect of their overall care, which may be affected by the morbidity associated with vascular access. Previous studies have tended, as we have, to focus on tangible outcomes, but other aspects such as the impact of repeated cannulation, the pain associated with this, convenience to the patient of rapid completion of dialysis therapy, significant bruising and bleeding need consideration in any subsequent powered randomised controlled trial to compare AVFs with CVCs.

### Limitations

Although fistula-failure rates have been reported, and CVC patients may go onto have an AVF fashioned, we did not look at patients who changed vascular access throughout the study. Unfortunately this information was not recorded within the database and may be a source of bias.

In addition, the retrospective nature of the study and reliance on information stored within local databases that may contain errors, limit the overall validity of the information. However, data was screened for spurious results and to the best of our knowledge, the data was correct. There were also a large number of patients with an unknown cause of death and aetiology of renal failure. This potentially confounds our findings with regards to the number of infection-related deaths. No data was available regarding the aetiology of renal disease or cause of death for the conservative cohort. This may have provided further insight into how this group compared to those who received dialysis. Furthermore, in this study we used eGFR≤10ml/min/1.73m^2^ to compare conservative and patients on dialysis, however in practice dialysis may not be initiated until below this value.

Selection bias of healthier patients for AVF creation is possible, with the assumption of better post-procedure outcomes. Whereas patients afflicted with multiple comorbidities and contraindications to surgery are often given CVC’s. Unfortunately data regarding mobility and frailty were not previously submitted to the UK Renal database due its subjective nature, and so have not been included within the study except as a comment as only 44 patients had data available. Of note, there is increased cardiac inefficiency in older patients and in those with chronic kidney disease due to multiple factors including the uraemic cardiomyopathy as a results of both volume and pressure overload in addition to other non traditional factors such as calcium phosphate balance and more recently FGF-23 [29]. Creation of an AVF may further exacerbate this and potentially lead to early demise.

In addition, the number of patients in each group was not equal and there is the possibility that the study was underpowered; perhaps in retrospect if the number of participants was greater, differences might have been even more pronounced and supported our conclusions.

Finally, the sample size for this study was relatively small, particularly the number of patients dialysing via an AVF, leading to a high risk that there was insufficient power to see any superiority from AVF. This is related to the age of the population selected. Although the number of patients starting renal replacement is increasing mainly due to the increase in the elderly cohort, the median age of dialysis patients in England in 2013 from the UK Renal Registry was 64.2 years (interquartile range: 51.0–74.6) (90% range: 31.3–83.9) [1]. There are perhaps two main reasons for the low numbers of patients over 80-years. The first is the high mortality in the chronic kidney disease population from cardiovascular causes and the second is potentially fewer referrals of this group of patients to the renal service. This particularly includes those with diabetes, who have multiple comorbidities. Data from the Renal Registry show that the proportion of patients aged 75-years and older was 17.4% in Wales, 16.4% in Northern Ireland, 15.8% in England and 13.7% in Scotland [1]. The figure for our unit was 15.7%. This study provides a unique insight into the predominantly Caucasian population of East Yorkshire. Furthermore, it provides an invaluable benchmark for future randomised control trials and will ultimately guide future best clinical practice.

In this population with multiple co-morbidities and possible significantly altered cardiovascular status, a randomized controlled trial, may be challenging and subject to ethical consideration given the possibility of harm. If we, for example, decide to allocate incident dialysis patients ≥80-years of age with similar comorbidities to either AVF or CVC, then we will actually harm those with altered cardiovascular status allocated in the AVF group. Exclusion of patients with altered cardiovascular status would result in a significant selection bias, since these diseases are a very common characteristic in this subgroup of patients.

## Conclusion

The choice of vascular access for those patients who choose dialysis ≥80-years remains a challenging decision. Age should not be a limiting factor alone, and comorbidities, level of frailty and informed patient preference should contribute to the overall decision. The decision to dialyse or not remains unknown in this age group, but perhaps dialysis did not offer a survival advantage in a significant proportion of the current cohort.

The literature advocates that where appropriate, an AVF should be attempted with better potential survival and lower morbidity from complications. However, our data suggests that there is no significant survival benefit over a 2-year period when comparing vascular access methods. The decision on whether and how (choosing the right vascular access method) to dialyse the over 80s is multifaceted and requires a tailored multidisciplinary approach [30]. This study provides an invaluable benchmark for future clinical trials to further guide best clinical practice.
